# Biobased Electronics: Tunable Dielectric and Piezoelectric Cellulose Nanocrystal—Protein Films

**DOI:** 10.3390/nano13152258

**Published:** 2023-08-06

**Authors:** Daniel Voignac, Shylee Belsey, Elisabeth Wermter, Yossi Paltiel, Oded Shoseyov

**Affiliations:** 1 Robert H. Smith Faculty of Agriculture, Food and Environment and Center for Nanoscience and Nanotechnology, The Hebrew University of Jerusalem, Rehovot 7610001, Israel; daniel.voignac@mail.huji.ac.il (D.V.); shylee.belsey@mail.huji.ac.il (S.B.); 2 Department of Applied Physics and Center for Nanoscience and Nanotechnology, The Hebrew University, Jerusalem 9190401, Israel; paltiel@mail.huji.ac.il; 3 Faculty 5, HSB—City University of Applied Sciences, 28199 Bremen, Germany; ewermter@stud.hs-bremen.de

**Keywords:** nanocellulose, proteins, bioelectronics, sustainability

## Abstract

Cellulose has been a go-to material for its dielectric properties from the onset of capacitor development. The demand for an energy storage solution continues to grow, but the supply remains limited and relies too often on fossil and mined materials. This work proposes a fully sustainable and green method with which to produce dielectric thin films made of renewable and degradable materials. Cellulose nanocrystals (CNC) made an excellent matrix for the dispersion of proteins and the fabrication of robust transparent thin films with enhanced dielectric permittivity. A range of proteins sources, additives and concentrations allowed for us to control the dielectric permittivity from *ε_r_* = 4 to 50. The proteins screened came from animal and plant sources. The films were formed from drying a water suspension of the CNC and proteins through evaporation-induced self-assembly. This yielded nano-layered structures with very high specific surface areas, ideal for energy storage devices. The resulting films were characterized with respect to the electrical, mechanical, piezoelectric, and optical properties to be compared. Electrically conductive (σ = 1.53 × 10^3^ S/m) CNC films were prepared with carbon nanotubes (CNT). The fabricated films were used to make flexible, sustainable, and degradable capacitors by layering protein-based films between CNC–CNT composite films.

## 1. Introduction

Most electronic devices rely on a variety of rare metals for components ranging from screens to batteries and processors. Most of these materials are fossil resources that need to be mined. Mining raises many environmental, working condition-related and health-based concerns and can be a source of geopolitical conflict [1,2]. While metals are sought for their electrical conductivity, another immensely important family of materials in the consumer electronics industry is fossil-derived polymers used for insulation and dielectric purposes [3].

This project seeks inspiration in naturally occurring nanomaterials and aims to contribute to the production of novel, sustainable, safe, and high-performance materials. Dielectric materials are widely used in many electronic devices, ranging from touch screens to capacitors. Capacitors store energy in the form of electrostatic potential energy. When an electric field is applied across a dielectric material, it causes charge separation and this is intrinsically related to the dielectric permittivity of the material, a value that quantifies the polarizability of the dielectric material. Paper is a pioneering dielectric material that has been used since the very early years of capacitors [4]. Paper’s building block is none other than cellulose. 

**Figure 1:**
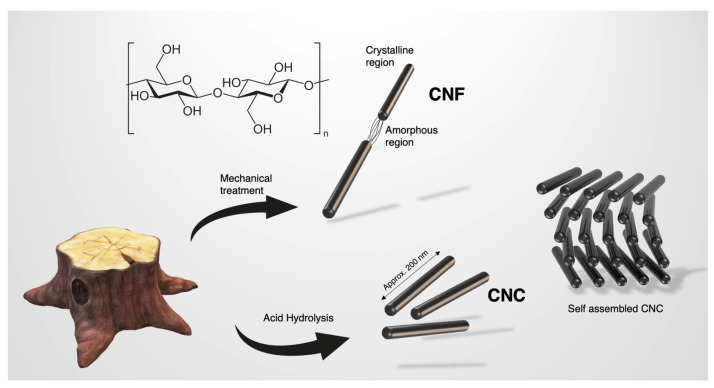
Cellulose and its nano scale derivatives, CNC and CNF. CNC has the unique ability to self-assemble in a chiral nematic arrangement upon evaporation of the water it is dispersed in [6].

Cellulose is the most abundant polymer on earth as it is a strength-providing constituent of the plant world. It makes up most of the plant cell wall and confers it its unique strength abilities. Cellulose has been very widely studied and the last decades have paved the way for the exploration of its nano scale applications. Cellulose is comprised of 2 glucose units bound together by a β 1→4 glycosidic linkage. This composition gives many available hydroxyl groups. Naturally, polymeric cellulose forms both amorphous chains and highly ordered crystals. Nanocellulose can refer to different materials and most often these are either cellulose nanofibers (CNF) or cellulose nanocrystals (CNC) as illustrated in Figure 1. The former are obtained via mechanical treatment, are typically made of two or more crystalline units bound together by amorphous cellulose chains, and yield nanofibers of micron length or greater. CNC is obtained using simple sulphuric acid hydrolysis. This cleaves the amorphous regions, which, after solvent exchanging against water, enables an aqueous dispersion of CNC, typically at percentages around 5 wt.% or lower. These crystals are approximately 200 nm in length and 5 to 10 nm in diameter. This depends on the source of the cellulose. Each plant will yield slightly different sizes of crystal and CNC can also be derived from some bacteria and even one animal, namely sea-borne tunicates [5].

CNC presents several key properties that make it highly relevant in this context. First and foremost, CNC self-assembles in strong transparent thin films upon the evaporation of water. This evaporation-induced self-assembly (EISA) results in the creation of highly ordered, anisotropic, stack of layers [7]. Due to the application of specific controls to the solution, and owing to the thermodynamics of drying, the crystals behave as liquid crystals and dry along a chiral nematic pitch. The aqueous suspension of CNC, along with the availability of many hydroxyl groups, makes it a very convenient platform for the integration of other nanoparticles or molecules into the wet phase. In the appropriate concentration and chemical conditions, these can be dispersed in the CNC-based layered dry matrix. This exploits the extremely high surface area generated by the EISA and creates the ideal substrates for nanoparticles. CNC presents outstanding mechanical properties as well. Single crystals in AFM have exhibited Young’s modulus of up to E = 150 GPa, whereas films, despite being two orders of magnitude lower, still display a very high performance around E = 4 GPa. This climbs to E = 8 GPa when the film is composite [8,9,10]. 

Aqueous CNC is thixotropic, making it very convenient for mixing in highly fluid states and casting in more viscous states, matching scalable fabrication methods for blade casting, such as doctor blade. Various parameters, such as pH and drying conditions, can be controlled to optimise mixing and nanocomposite generation [11,12]. pH can be tuned to match the stability requirements via either salt titration or conventional dialysis methods. Many functionalisation methods are also available, with the most popular being TEMPO oxidation [13]. When combined with residues from sulphuric acid hydrolysis, CNC’s surface is negatively charged, and the crystals exhibit amphiphilic behaviour, excellent for the formation of Pickering emulsions. Methods for complete surface modification have also been developed, including rendering CNC hydrophobic [14]. 

This brief introduction to CNC shows how it appears to be an excellent candidate for use in a sustainable platform for low-cost nanocomposites. This work seeks biomaterial additives with which to increase and tune the dielectric permittivity of CNC-based self-assembled thin films. Proteins are chains of amino acids, with different orders of structure and many functional groups. Most amino acids exist as chiral zwitterions and are therefore highly polar and highly dielectric. Two families of protein are introduced in this preliminary study: bovine serum albumin, a staple animal-derived protein very commonly used in laboratories, and a series of plant derived proteins, which are more scalable, environmentally friendly, and vegan. 

Biomaterials and their dielectric properties are gaining interest in the academic literature. Fibre-reinforced plastics make up a large family of advanced materials and the dielectric properties of biofibre-based composites have been reviewed [15]. Proteins are a subset of biomaterials. While they are mostly studied for their biological properties, there is growing interest in evaluating the other intrinsic properties of proteins in the field of materials science. They are complex polymers that are renewable and degradable, while also being highly functional in both chemical and physical terms. Some of the proteins evaluated in this work are among the most studied proteins in the animal and plant kingdoms [16]. Zein, a derivative of maize (corn, one of the largest crops in the world after sugarcane), has been used as a raw material in various industries. John et al. have applied zein to modified flax fibres to create flax-reinforced polypropylene composites for use in dielectric applications [17]. CNC–zein composites have been evaluated for their use and oxygen and water vapor barrier properties [10]. This work aims to investigate protein-reinforced CNC composites for use in dielectric applications. 

The resulting films will be broadly characterised with respect to their mechanical, optical, chemical, structural, electrical, dielectric, and piezoelectric properties. This preliminary characterisation will help to map out a spectrum of applications for such materials. In order to illustrate the potential impact of this technology, fully degradable, flexible capacitors will be described. These capacitors have dielectric cores and are made up of CNC–protein films and two electrodes made of simple composites of CNC and carbon nanotubes (CNT). Carbon nanotubes were selected as a staple non-metallic conductive nanoparticle. These devices could bring a novel perspective to various reports of nanocellulose-based composites for use in capacitive and/or conductive applications [18,19,20].

## 2. Materials and Methods

### 2.1. Materials

Cellulose nanocrystals (CNC) were kindly supplied by Melodea Ltd. (Rehovot, Israel) as a suspension of 3.6 wt.% in water at a pH of 4.2. All plant-based proteins were kindly supplied by Egg’n’Up Ltd. (Rehovot, Israel). Zein, sodium hydroxide (NaOH) flakes, bovine serum albumin (BSA), 1,2,3,4-butanetetracarboxylic acid (BTCA) and sodium hypophosphite (SHP) were purchased from Sigma-Aldrich. Carbon nanotubes were supplied by Tortech Ltd. (Ma’alot Tarshisha, Israel)

### 2.2. CNC Protein Preparation

CNC was diluted to 2 wt.% with double-distilled water (DDW) and titrated with dropwise addition of 100 mM NaOH solution in order to achieve a final pH of pH = 7. In all protein-based films, 2 mg/mL of protein was added in a powder form to achieve 1:10 dry mass ratios to CNC. For crosslinked films, BTCA was added at 3 mg/mL of 2 wt.% CNC and SHP 0.1 mg/mL of 2 wt.% CNC [21]. (CNC with crosslinkers is abbreviated CBS in the text.) 

The solutions were then sonicated for homogeneous mixing with a Q500 Sonicator (QSonica L.L.C, Newtown, CY, USA). Sonication was performed using a pulse of 1 s on and 1 s off for a total time of 10 min at an amplitude of 40%. The solutions were then centrifuged at a relative centrifugal force (rcf) of 5000× *g* for 5 min at 22 °C to check for the absence of any pellet formation and remove air bubbles. 

Solutions were then cast on a polyimide substrate via a doctor blade method (Elcometer knife applicator) that fixed the blade 3 mm above the substrate. The substrate was taped on its four edges with autoclavable tape to create sufficient surface tension to maintain the solution at a homogenous height during drying. Samples were left to evaporate in a dehydrator with a central air flow kept at 35 °C. The evaporation-induced self-assembly (EISA) allowed the delamination of a self-standing film after overnight drying. 

### 2.3. CNC Carbon Nanotube Film Preparation

The preparation of the CNT films used as electrodes for the all-CNC-based capacitors was akin to the above protocol described for CNC–protein film. CNT was added at 2 mg/mL to a 2 wt.% CNC solution. For crosslinked CNC–CNT (i.e., CBS–CNT), the same concentration of BTCA and SHP as above was added. The solution was sonicated in 4 series of 10 min total sonication time. Centrifugation was performed between each sonication step to assess dispersion. The sonication was stopped after the 4th sonication series, the point where satisfactory solution homogeneity was achieved. Given the black colour of the solution, the stability could not be assessed by looking for a pellet. Therefore, the solution was considered homogeneous when it could pass through a pipette tip. Solutions were cast in the same way but dried in an oven at 65 °C to achieve faster drying and activation of the crosslinking.

### 2.4. Mechanical Analysis 

Mechanical tensile tests were performed using an Instron 3345 universal testing machine (Instron, Norwood, MA, USA). The films, cut into pieces of 4 mm width and 20 mm gauge length, were tested in a tensile mode using a 100 N load and a tensile speed of 2 mm/min. Young’s modulus, the elongation at fracture, and ultimate tensile strength were determined from the resulting tensile stress curves. Toughness was computed as the area under the test curve using a trapezoidal method. All samples were bent along rods of various radii and no samples broke when bent up to a 2 mm bending radius. 

### 2.5. Electrical Characterisation 

#### 2.5.1. Resistivity 

The resistivity of the thin films was measured using a simple 4-point probe method. The 4 probes were equally spaced on the surface of the film (5 mm apart). A constant current was supplied across the two outer electrodes and the voltage between the inner electrodes was recorded. The resistivity was computed using Equation (1).

(1) $$\rho =t\;\rho_{s}=t\times \frac{V_{in}}{I_{out}}\times \frac{\pi }{\ln\left(2\right)}\equiv \left[\Omega \cdot sq\right]$$

where ρ is the resistivity, *t* the thickness of the film, $\rho_{s}$ the sheet resistance, $V_{in}\;$the voltage between the inner proves, and $I_{out}$ the current supplied across the outer probes. For all the CNC and CNC–protein films, a constant 10 nA DC current was supplied across the sample using a Keithley 6221 DC and AC current source (Keithley Instruments, Cleveland, OH, USA).

#### 2.5.2. Dielectric Permittivity

The dielectric permittivity of the composite films was measured by constructing a parallel plate capacitor. Capacitance was measured with a DMM6500 6 1/2-digit multimeter (Keithley Instruments, Cleveland, OH, USA). A mount consisting of two identical holders for copper (Cu) plates cut into 10 mm × 10 mm was 3D printed, with slits included to fit the Cu plates parallel to each other. Contact was made using two clips on the system (cf. Figure S1). The dielectric permittivity was computed from the capacitance as described Equation (2), where *C* is the capacitance in Farads, $\varepsilon_{0}$ is the dielectric permittivity of vacuum (taken as *ε*_0_ = 8.854 × 10^−12^), ε is the absolute permittivity of the material, $\varepsilon_{r}$ is the relative permittivity of the dielectric core, *d* is the distance between the electrodes in meters, and is *A* the surface area in m^2^.

(2) $$C=\frac{\varepsilon A}{d}=\frac{\varepsilon_{r}\varepsilon_{0}A}{d}↔\varepsilon_{r}=\frac{Cd}{\varepsilon_{0}A}$$

#### 2.5.3. Piezoelectric Behaviour

The influence of protein introduction on the piezoelectric behaviour of the resulting films was assessed using a simple electro-mechanical setup. A Cu parallel plate holder was prepared via a similar method to that seen in the previous section and placed without clips in an Instron 3345 with compression testing heads. A cyclic loading of increased force (1, 2, 3, 5, 10 and 25 N) was applied at a 250 mm/min rate in cycles of 5 compressions. The Cu plates were connected to a Keithley DMM6500 6 1/2-digit multimeter and the resulting DC voltage peaks were recorded, averaged and compared. Three sets of 5 cycles were performed on each film and 3 films of each composition were tested. 

### 2.6. Chemical Analysis

The infrared spectra of each composition were recorded for reference using a Nicolet 6700 FTIR-ATR Spectrometer (ThermoFisher Scientific Inc., Waltham, MA, USA) with a smart iTX Accessory. Sixteen scans were taken per sample from 4000 to 400 cm^−1^ at a resolution of 4 cm^−1^. The visualisation and collection of the collected data were performed using the OMNIC^TM^ Series software (ThermoFisher Scientific Inc., Waltham, MA, USA).

### 2.7. Structural Analysis

The nanostructure of the films was revealed via cross-sectional scanning electron microscopy. Samples were prepared by submersion in liquid nitrogen in which they were fractured by impact. The fractured edges were coated with gold–palladium (Au:Pd −80:20) using a Q150T ES sputter coater/turbo evaporator (Quorum Technologies Ltd., Lewes, UK), 60 s with a DC current of 12 mA. Due to the conductive properties of CBS + CNT, only CNC, CNC + BSA and CNC + sunflower were sputter-coated. These four samples were imaged with a JSM-7800F scanning electron microscope (JEOL Ltd., Akishima, Japan). Thanks are due to Dr. Einat Zelinger for technical assistance. CNC–BSA and CNC–sunflower were the only two CNC–protein films selected as illustrations of animal-based and plant-based protein. 

### 2.8. Optical Analysis 

Optical analysis of the films was performed using a Thermo-evolution 300 UV-VIS spectrophotometer, (ThermoFisher Scientific Inc., Waltham, MA, USA). The transmittance spectra of the films were recorded in the range from 190 nm to 800 nm. 

### 2.9. Capacitor Fabrication

Simple capacitors were built by stacking three CNC-based films. The two outer films acting as electrodes are CNC + CNT films cut into 15 mm × 10 mm areas. The dielectric cores were cut from the CNC protein or pure CNC, respectively, as a control. The core dielectric film was cut into 11 mm × 11 mm pieces to make sure the two electrodes were insulated. The films were stacked and 25 μL of the aqueous solution used to cast the core dielectric films was used as a binder to ensure contact. 

## 3. Results

**Figure 2:**
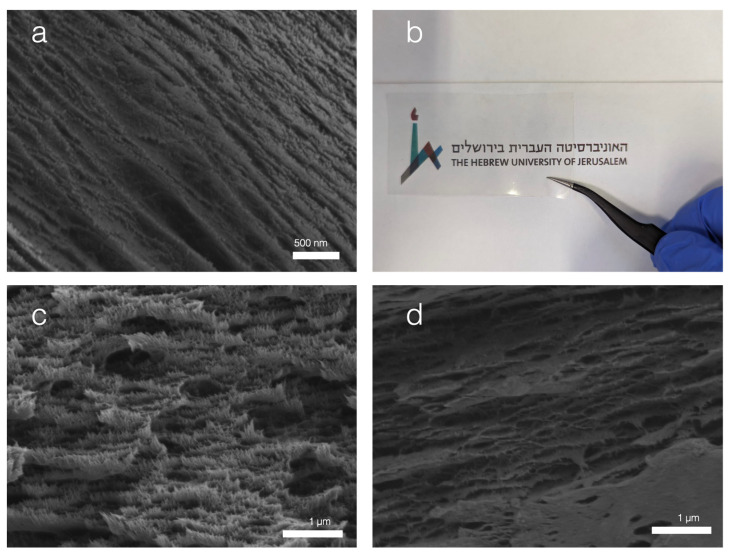
Structure and morphology of CNC and CNC protein films. (**a**) Cross-sectional SEM micrograph of a pure CNC film displaying a typical layered structure. (**b**) Self-assembled CNC–aquafaba film. The film is self-standing and optically transparent, and all CNC–protein films have a similar appearance. (**c**) Cross-sectional SEM micrograph of a CNC–BSA film, where layered structure is conserved. (**d**) Cross-sectional SEM micrograph of a CNC–sunflower seed protein showing that the CNC’s self-assembly is also conserved, although its crystals are less well defined.

All CNC–protein films appeared the same at the macro scale, with slight taints ranging from yellow to grey. The optical properties are presented later to quantify these colour difference. The taints of the film corresponded to the colours of the dry protein powders. Since all the CNC–protein films made at the same 1:10 ratio resembled a pure CNC film (cf. Figure 2c), a morphology analysis was performed to confirm that the naturally occurring self-assembly of the CNC remained the main driving mechanism during evaporation-induced film formation. 

The scanning electron microscopy images showed in Figure 2 compare the morphology of a pure CNC film and two CNC–protein films. An animal-based protein (bovine serum albumin (BSA)) and a plant-based protein (sunflower seed) were selected as representatives of their respective categories. Both protein films showed the same layered structure as the pure CNC. The resolution of the CNC crystals differed slightly, and the less well-defined crystal arrangement may point to amorphous proteins sitting in between the crystals. 

**Figure 3:**
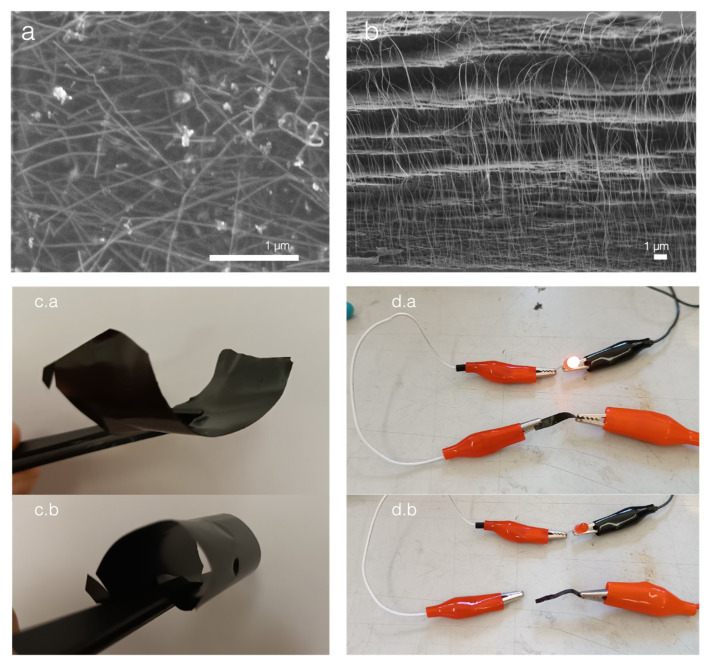
Structure and morphology of the CNC–CNT composites used as electrodes. (**a**) SEM micrograph of a CNC + CNT film uncoated, white tubes are CNT, and scale bar reads 1 μm. The tubes cover the surface and seem to overall interconnect, justifying the overall conductivity of the sample. The bright particles are assumed to be dust that settled on the film during evaporation. (**b**) Cross-sectional SEM micrograph of the CNC + CNT uncoated, scale bar reads 1 μm, the background shows the characteristic CNC layered self-assembly while the white hairy filaments coming out of the cross-sectional plane are the CNT, which is more conductive and thus brighter. The conservation of the CNC-driven self-assembly suggested anisotropy. (**c.a.**) shows the final self-standing film in a bent mode and (**c.b**) in a rolled mode. Images (**d.a**,**d.b**) show that the film can be used to close a circuit and is conductive enough to switch on a red LED.

The CNC–CNT films were studied separately. The carbon nanotubes are black in appearance and dominate the color in the composite film. The films formed were very robust and electronically conductive, with a surface conductivity that measured at σ = 1.53 × 10^3^ S/m. This is illustrated in Figure 3c,d where the films close a circuit to light a simple LED. Their nanostructure was also more complex than that of the CNC–protein films. Since the CNT used were already conductive, SEM was performed on uncoated samples to enhance the contrast between the molecules. Unlike most conductive nanocomposites, elemental analysis such as EDS mapping was not relevant as carbon conductors cannot be differentiated from the carbon backbone of the CNC. With conduction occurring on the film’s surface and across the film along different axes, both top-view and cross-sectional imaging was performed. The top-view analysis reveals a random arrangement of overlapping CNTs. The bright fibers correspond both in dimensions and electron count to what is expected from the CNT used. The cross-sectional analysis shows the typical layered structure expected from a CNC film, with hair-looking bright fibers observed out of the cross-sectional plane. The cross section was prepared via the impact fracture method in liquid nitrogen. While this method can easily produce a clear cut in the CNC arrangement, it was probably insufficient to fracture the CNTs. Thus, the analysis of micrograph 3b. suggests that the CNC still drives film formation. CNTs overlap one another and sit in between layers of CNC as well. The maintained presence of CNC layers also points to the anisotropy of the film, which can be further exploited in different electrical applications. A key limitation of CNTs are the dominating Van der Waals forces that cause agglomeration and poorer performance. The essential measure taken to overcome the Van de Waals forces was the use of ultrasonication for the attainment of stable dispersion. The sonication time was 4 times longer than that required for the CNC–protein. The CNC help to prevent the reformation of Van der Waals interactions between the CNT by intercalating between them and may form Pickering emulsions [22]. The stability of the ultrasonicated mix was determined by centrifugation and checking for lumps in the solution. This method still does not entirely overcome the Van der Waals forces. There may be localised small agglomerates that are not perceptible by eye. The composition was selected empirically when it produced a film with a resistance below 1000 Ω. Other methods have been employed to improve the mixing and these are dependent on the viscosity of the polymer CNTs are dispersed in, such as the three-mill roll proposed by Khalid et al. in 2022 [23].

The main differences in appearance between the pure CNC film and the CNC–protein films were slight taints in colour. To quantify these differences, simple UV-VIS spectroscopy was used. To compare this general observation, the transmittance across the visible range (400–800 nm) was averaged and the results are reported in Table 1. BSA, Aquafaba and pea films had the closest appearance to pristine CNC. With an average transmittance above 80%, these films were considered optically transparent, while all others except year protein were only translucent, yielding an opaque film [24]. The value for aquafaba even surpasses the recorded CNC transmittance. Further investigation would be necessary to confirm whether the aquafaba has a role in improving the transparency of the CNC. Aquafaba is a protein that is found in the water used after boiling legumes, and as such it is a water-soluble protein [25]. The optical properties of the composite CNC–protein films may be directly related to their water solubility. CNC is amphiphilic and is known to form Pickering emulsions [22]. These nano- to micro-sized emulsions may be imperceptible to the naked eye in the wet phase and be the cause for the transmittance changes. The deviations in Table 1, as well as the graphs in Figure S3, suggest that the transmittance does not dip at a specific wavelength. Rather, it appears that the reduced transmittance is uniform across the visible range. ChickP G and yeast protein films transmitted less than 50% of the light across the visible range. These were the least clear films, with visible agglomerates formed on the film, further suggesting the direct influence of water solubility. The sunflower seed film has the highest deviation, the explanation for which is graphically detailed in the supplementary information, but transmits much more in terms of higher wavelengths, a fact that explains its warmer taint (cf. Figure S4). The crosslinked BSA film (CBS BSA) transmitted an average 6% less light across the visible range. This could originate either from pH changes in the wet phase due to the introduction of the carboxylic acid causing damage to the BSA [26], or from the crosslinked network formed by the acetylation of cellulose’s hydroxyl groups.

The UV transmittance results are presented in Figure S2 in the supplementary information. All CNC protein films reveal a shift in UV absorbance range when compared to pure CNC. 

**Figure 4:**
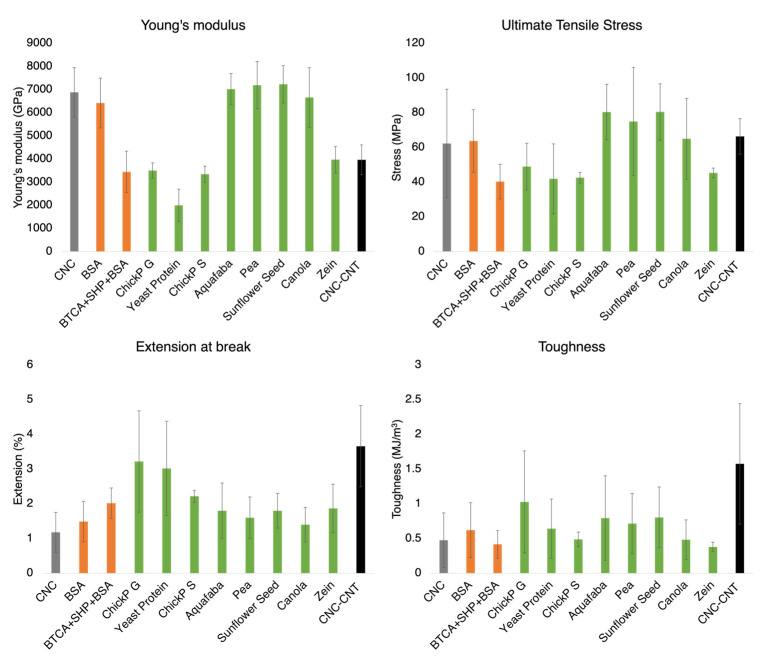
Summary of the tensile testing of the different CNC-based films in this evaluation. The values computed from the stress-strain curves are, top to bottom, left to right, Young’s modulus E, in MPa, ultimate tensile strength (UTS, in MPa), extension at break in % and toughness in MJ/m^3^ (computed as the area under the curve using a trapezoidal method).

No specific differences were felt in terms of the dry films’ mechanical performance. All the films were able to bend to a 2 mm bending radius (cf. Figure S6). This homogeneity was also observed in the tensile testing of films. These tensile tests are summarized in Figure 4 using Young’s modulus, toughness, ultimate tensile stress (UTS) and extension at break. The graphs are plotted in Figure S5. All results are in the same order of magnitude, but key differences can be noted. The ChickP and the yeast protein films are the bottom performers in terms of Young’s modulus and UTS, which correlates with the poor optical transmittance and the visible agglomerates formed on the film. The poor mixing can weaken the matrix by introducing multiple crack propagation and stress concentration points. Surprisingly, none of the films seem to outperform pristine CNC in strength, with several CNC–protein films such as BSA, aquafaba, pea, sunflower and canola yielding comparable results for Young’s moduli ranging between E = 6.5 and 8 GPa and UTS ranging between 60 and 80 MPa. The high standard deviations will be discussed in the following section. The CNC–CNT composite film stood out in terms of toughness. The CNT’s contribution to this improved toughness may correlate to the behaviour that resulted in micrograph Figure 3b, where the out-of-plane nanotubes appear to not have broken in the liquid nitrogen fracturing. The crosslinked BSA film performed poorly compared to its non-crosslinked equivalent. BTCA–SHP crosslinking had previously been used to strengthen CNC films, pointing to the adverse effect of the crosslinkers on the protein in the aqueous phase, and later perturbated the CNC-driven self-assembly. Overall, the films still perform excellently when compared to most synthetic plastics.

**Figure 5:**
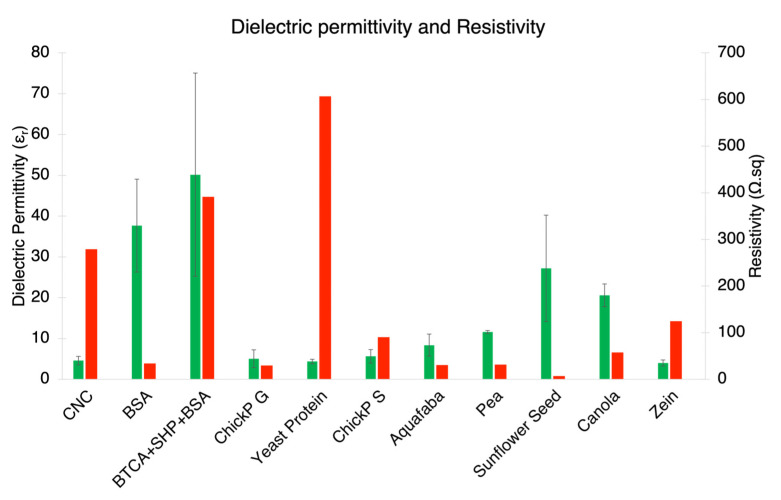
Relative dielectric permittivity and resistivity of the different composites fabricated. Green bars refer to permittivity (left axis) and red bars to resistivity (right axis). All proteins were introduced at a 1:10 dry mass ratio to CNC. Labels refer to the protein introduced in the film.

The main subject of study in this work is the impact of proteins on the dielectric properties of CNC films. The measurement of dielectric permittivity was used in a very simplified parallel plate capacitor model, which will be discussed in the following section. The dielectric permittivity of the films ranged between *ε_r_* = 4.0 to 50.1, where CNC was measured at *ε_r_* = 4.6. The graphical plot of these values in Figure 5 shows high deviation, which can be mostly attributed to setup errors and factoring in humidity as discussed in the discussion section. The resistivity, plotted onto the same graph (right axis, in red), ranged from 7.0 Ω·sq for the CNC–pea composite film up to 607.0 Ω.sq for yeast protein. This was measured using a simplified 4-point probe measurement and the surface may be different to the core of the film due to its interface with air during drying and the possible sinking or floating of impurities in the wet phase. The CNC–CNT film had a resistivity of 6.52 × 10^−4^ Ω.sq, which equates to 1.53 × 10^3^ S/m.

**Figure 6:**
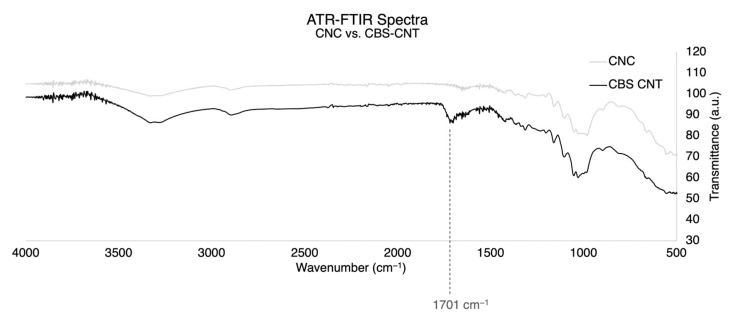
ATR-FTIR spectra of a pure CNC film vs. a CBS–CNT film. The latter refers to a film made of 1:10 CNT to CNC ratio with BTCA as a crosslinker and SHP as the catalyst for the crosslinking to occur. The highlighted peak at 1701 cm^−1^ confirms the crosslinking in which the carboxylic groups of the BTCA and the available hydroxyl groups in the CNC formed a new acetylated structure. The transmittance is read in arbitrary units (a.u.) as relative to the background and environment of the measurement.

The chemical analysis of the films was performed by taking an FTIR spectrum of each film in attenuated total reflectance (ATR) mode, which is suited for sampling films. This characterisation allowed us to take a chemical fingerprint from each of these novel composites and also enabled the tracking of the crosslinking reaction with BTCA. Figure 6 illustrates the formation of a peak at 1700 cm^−1^, which is characteristic of the expected acetylation reaction when crosslinking the carboxyl groups of BTCA with the hydroxyl of cellulose to form ester bonds. The spectra are detailed in Figure S7 in the supplementary information.

**Figure 7:**
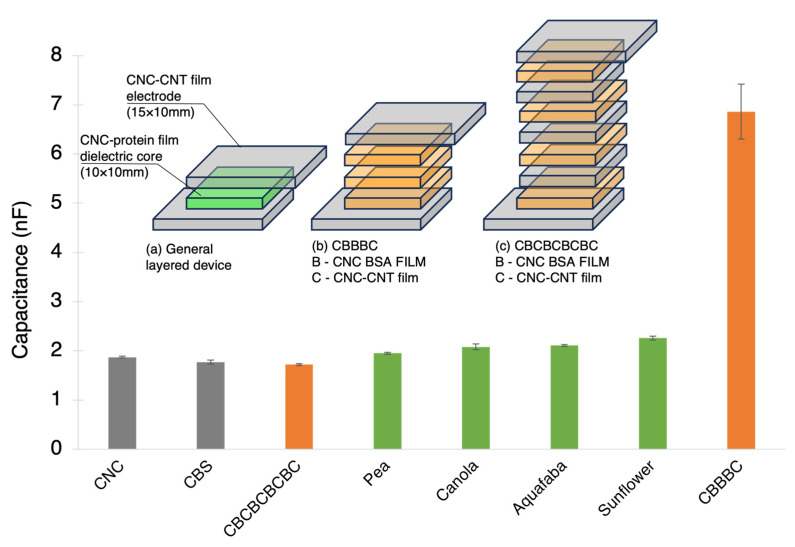
Capacitance of sandwiched devices constructed from CNC–CNT films as electrodes and CNC–protein films as dielectric cores. The general device is illustrated in (**a**) where top and bottom electrodes are made longer on each side for contact with multimeter. Grey bars are reference CNC and crosslinked CNC (CBS) cores, green bars refer to plant-based dielectric cores and orange bars refer to animal-based, (BSA)-based CNC films. CBBBC refers to a threefold stack of BSA core and CBCBCBCBC refers to a fourfold stack of BSA where each BSA film is separated by a CNC–CNT electrode film, as illustrated in (**b**,**c**).

The fabrication of both dielectric CNC–protein films and conductive CNC–CNT films allowed the very simple fabrication of flexible, sustainable, and degradable capacitors made from a sandwich structure of conductive and dielectric films. These simple devices are a proof of concept for an industrial application of such novel composites. The capacitance of these devices is illustrated in Figure 7. 

These showed a slightly increased capacitance where the device with a CNC core yielded 1.87 ± 0.02 nF and a CNC–sunflower core device increased this value 2.26 ± 0.04 nF. These values are very preliminary and will be discussed in the section below. Proper cyclic voltammetry should be performed in order to further investigate these values. The devices were also later polarised for 5 min under 10 V of applied voltage across the two outer CNC–CNT electrode films. The voltage discharge was measured after the 5 min poling and the decay was fit to estimate the time to discharge to 1.5 V and the time to total discharge which are summarised in Table 2.

The discharge curves (cf. Figure S8) are also preliminary results. However, the striking differences in the sustained discharge when the proteins were introduced call for further investigation and for cyclic voltammetry in particular. The differences in discharge rates are non-linear. The CNC–pea dielectric core proved best for sustained discharge to 1.5 V, but the CNC–sunflower-seed-containing film was the slowest to completely discharge. The discharge data were collected for a minimum of 5 min and a logarithmic fit was used to predict the total discharge rate. These data are available in the supplementary information (cf. Figure S8 and Table S1).

**Figure 8:**
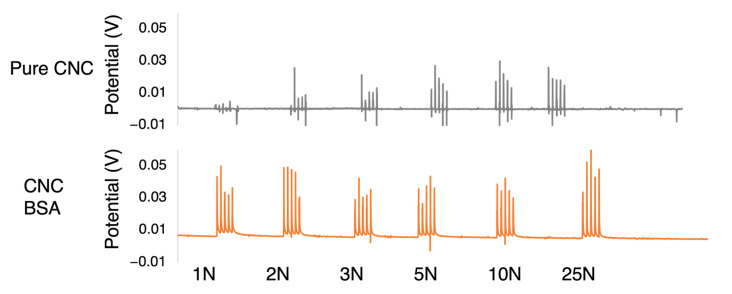
Potential measured across films upon salves of 5 compressive impacts. A pure CNC film was taken as reference and compared to a CNC–BSA film. The CNC–BSA seems to have generated a higher potential and with a lower threshold of impact force required.

Finally, a simple impact test was designed to evaluate the change in the piezoelectric properties of the CNC film. Cellulose is a known piezoelectric material and the self-assembled structure can be extremely beneficial for harvesting electrical energy from mechanical deformation. Current yields of energy conversion have been very low, and the test was performed on one of the CNC–protein films (CNC–BSA) to empirically assess whether protein could have a role in improving the piezoelectric behavior of CNC films. A serial increase in applied force was recorded from 1 N to 25 N with various different increments. Each impact was repeated 5 times in salves of 250 mm/min displacement of the force transducer. The resulting potential was recorded and is reported in Figure 8. 

## 4. Discussion

This work shows that CNC–protein self-assembled composites can be seen as a platform for the rapid, cheap, safe, and simple fabrication of complex dielectric nanocomposite films. The films are strong, flexible, transparent, or translucent and are made from biodegradable materials. This work focused on looking at a range of proteins, and two main angles will be discussed in this section to comment on and complete our presentation of the above results. The first pertains to the intrinsic limitations posed by CNC and how some of these can be overcome. The second angle of discussion relates to the limits of the high-level characterisation methods employed and will suggest tools for a deeper analysis of these composites.

One of the main takeaway points in the results section is that proteins enable the editing of CNC films’ dielectric properties and can be used to enhance the dielectric permittivity. The results have relatively high standard deviations that may originate from different steps in the process as well as the assembly of the material. The self-assembly of the CNC is mainly driven by the shape, size, and surface charge of the CNC. These factors are highly dependent on the sourcing and preparation method [27]. In 2016, Reid et al. published an initial benchmarking of CNC covering both lab-made and industrially purchased CNC. This was followed by a complementary review by Delepierre et al. in 2021 [27,28]. These reviews did not include a survey of the dielectric properties but the variations in liquid crystalline self-assembly already suggest that such a benchmarking analysis would prove useful to the scalable and controlled manufacture of CNC-based dielectric substrates. The size of CNCs can vary greatly depending on their origin and less conventional sources such as tunicates have been shown to provide much higher aspect ratios [29]. The packing of the CNC in the cross-sectional electron micrographs of Figure 2 and Figure 3 illustrates well the influence of the self-assembly on the dielectric properties. Indeed, it appears that these self-assemblies dictate the interfaces electrons will have to cross under the polarisation of the film. The anisotropy can be further studied to assess if it can be exploited in situ. The dielectric permittivity and resistivity should be tested in different directions and may yield applications in the large-scale integration of different devices in complex 3D arrangements. This point also highlights the limitations in the resistivity measurements. The 4-point probe method used assumes a uniform behavior across the thin film and does not account for anisotropy. This model also does not account for surface impurities, such as those seen in the top-view micrograph of a CNC–CNT film in Figure 3a.

CNC–CNT films were not the object of this study but were chosen as a simple, cellulose-based metal-free electrode. The CNC–CNT composite system has been the object of many studies [30,31,32,33]. The immense range of CNTs and the influence of many parameters, including size, size distribution, chirality, surface functionalisation, and more, will influence the percolation of CNT in CNC [34,35,36] and could constitute the grounds for a standalone publication. For this project, the aim was to achieve a simple film with an arbitrary resistance below the 1000 Ω threshold. Several concentrations were tried and the 10 wt.% to dry CNC mass was empirically found to pass this set threshold. 

The fabrication methods and conditions of EISA can also influence its measurements and applications. CNC grants the ability to form low-cost nanotechnology as it provides templating for nano-scaled features and solid-state separation of the additives once the film is dried. The rheology of the wet phase is very convenient in efforts to form films and matches the requirements of upscaled manufacturing. The thixotropic behavior of CNC allows for the easy introduction of additives into the fluid aqueous suspension while high-energy mixing (e.g., here, ultrasonication) is provided. However, it also enables scalable industrial casting methods, such as the doctor blade technique, for a controlled casting height during the EISA phase where the suspension displays a gel-like viscosity. A continuous line of casting from the aqueous phase to reel-rolling of dry self-assembled film can pave the way for industrial use. Exerting high levels of control over airflow, humidity, temperature, and filtration can also prevent some of these impurities from settling on the surface. 

The central investigation of this work is, of course, the proteins themselves and their influence on dielectric properties of the system. Proteins are complex chains of amino acids and the choice of protein for this survey work was driven by ease of access to said protein and the desire to screen preliminary results. The first protein used for this aim was BSA as it is a very well-known water-soluble protein, widely available at the lab-scale. The encouraging preliminary results led to the pursuit of a more sustainable approach. Following the trends of the food industry, plant-based proteins were sought for use in this evaluation. These lower the environmental footprint and do not pose any animal suffering issues. Throughout this research, the proteins were approached as raw materials and, other than titrating the pH of the CNC suspension to pH 7, no measure was taken to conserve any of the complex properties of proteins. The results can be further developed by seeking correlation between the amino-acid sequences and trying to assess the role of primary, secondary and tertiary structures in influencing dielectric properties. The powders were added as supplied regardless of their purity. It remains unclear whether the proteins maintained any tertiary structure after the application of the ultrasonication treatment used in this fabrication method. This may be assessed later using crystallography methods such as SAXS to resolve the CNC–protein films against known protein structures or SDS-PAGE gel electrophoresis. Should the mixing be too destructive to the proteins and negatively affect the dielectric permittivity, other approaches can be used, including weaker sonication or simple rotative mixing for longer periods of time when the protein is introduced just after sonication of the CNC. Adjusting the acidity of the 2 wt.% CNC solution to pH = 7 was performed to generate uniform conditions across the CNC–protein (non-crosslinked) films and to maximise the chances of maintaining stability [37].

Modelling is a fundamental step in electronics design. It is required for optimal design, precise engineering, and safety compliance. This work shows that the top-down approach is extremely limited for application to these types of composites. Electron microscopy confirms the presence of a complex morphology with CNC’s typical layered structure. The anisotropy introduced at the nanoscale should translate to anisotropy in the electronic behaviour of the films and this suggests that the modelling of the materials should be at least bi-dimensional, assessing the x–y plane and the *z* axis (along the stacking) as two different contributions. The complexity does not stop there, as proteins are complex chains of amino acids where each position in the chain can be 1 of 22 possible molecules, each bearing a different dielectric permittivity. Modelling the primary chain of amino-acids may also prove insufficient for the precise modelling of the proteins as they are known to conform in complex secondary and tertiary arrangements, thus increasing the combinatorial work required to model them and the complexity of the bottom-up modelling. Amin and Küpper recently described a semi-empirical model for the computation of proteins from the protein data bank (PDB), which has over 150,000 entries [38].

As contextualised by Huang et al., there are 20^200^ possible amino-acid sequences for a 200-residue protein [39]. The so-called “AI-revolution” of the past decade is bringing more advanced computational tools, such as DeepMind’s Alpha Fold [40]. Recently, Ni et al. reported on an attention-based diffusion model de novo protein design generator [41] based on secondary structure input. Many other methods have been proposed, including some that are very specific to the dielectric properties of proteins [42,43]. These recent advances may pave the way for recombinant or de novo proteins that may be designed bottom-up to suit specific dielectric or even conductive applications and bind to biopolymers such as cellulose through cellulose-binding domains. The growing field of synthetic biology can provide realistic pathways for the bio-manufacturing of these highly specific, high-performance “industrial proteins”. Such methods are costly, but the highly specific and tailored properties that could be achieved with such systems could tap into cost-independent niches of the dielectric market looking for high-performance. 

The mechanical properties of the films are within the same order of magnitude and correlate with the existing literature on CNC–protein films [9,10]. The magnitude of the error bars in Figure 4 are mainly attributed to the size of the strips tested, their preparation and conditioning. The stress in MPa is computed by dividing the force measured by the cross-sectional area of the film tested. With the width in mm (10^−3^ m) and the thickness in tens of μm (10^−6^), the influence of slight variations in thickness and width can be very high on the stress measured. The samples are also brittle, and their fracturing depends on the cutting methods. An irregular scissor cutting can introduce cracks, which act as propagation sites and accelerate fracturing. Such stress concentration sites can also originate in defects such as impurities that develop during drying or bubbles. In addition, the pre-conditioning of the film to a specific relative humidity or lack thereof may also improve the deviation. Environmentally controlled testing can be performed together with more detailed mechanical testing in dynamical mechanical analysis (DMA) setups.

The variations in dielectric permittivity may arise from setup-related irregularities such as contacts. The parallel plate capacitor model was chosen for its simplicity and high throughput but is dependent on many factors. The copper plates were regularly sanded with P150 grit sandpaper to remove the oxide layer. However, this also increased the surface roughness and created subsequent air gaps between the electrodes and the dielectric film. These was partially negated by applying force. This was performed using 3D printed clamps, relying on the elastic modulus of poly-lactic acid from which the copper plate holder and clamps were printed. However, given that cellulose is also piezoelectric, applying mechanical force also generates noise in the resulting signal. 

All proteins were introduced in a 1:10 ratio and all films were cast in the same conditions at the same wet-phase thickness. An exception to this was made for crosslinked films, which we intended to show that some manipulations can be performed to tune biodegradability, and CNT-containing films, used for their electronic conductivity. This research attempt to simplify characterisation in order to assess films as bulk dielectric substrates yielded in high standard deviations in results, and also highlighted different levels of complexity. The other axiom taken in the experimental section of this work was to fabricate and test in ambient nominal conditions and thus assess the robustness of such composites in relation to realistic use cases. 

One of the principal limitations in this experimental design is not considering the effect of confined water. The dielectric permittivity is a factor of temperature and relative humidity. Water (H_2_O) is a polar molecule and CNC is highly hygroscopic. Water molecule will adsorb to the surface of the crystals. In 2019, Lunev et al. reported an evaluation of hydrated water as a structural component of CNF [44]. Dielectric spectroscopy was performed with the preconditioning of the films to various humidity conditions and was run across a wide range of AC frequencies (10^−2^ Hz–10^6^ Hz). This present work does not include vacuum or controlled humidity controls as it is rather a preliminary screening of nominal working conditions for applications such as disposable/degradable electronics. In future a more controlled dielectric spectroscopy analysis may be performed to deepen the understanding of the role of the protein as well as refine the permittivity measurement and obtain complex permittivity in order to extract the dielectric loss factor as well, another key missing parameter in this study. 

The piezoelectric assessment was limited to an indirect empirical recording on a single sample. While the method was adapted from Choi et al., who tested nanocellulose-ceramic films for piezoelectric performance [45], a more precise computation of the piezoelectric coefficients matrix should be further performed. The CNC–BSA generated a higher potential upon impact and seems to have reached a plateau, starting at lower forces, suggesting it generates a more sensitive and efficient composite film than pure CNC. Different mechanisms can be hypothesized for this improved behavior. The protein may have intrinsic piezoelectric properties. It may cause synergistic effects such as coupling or other amplification and resonating behaviors. The visual aspect of the cross-sectional micrograph in Figure 2c also suggests that the protein can have a mechanical effect by forming a “glue” around the crystals. These effects could be further assessed by atomic force microscopy measurements combined with varying concentration and improved bulk film piezo characterisation. The dampening of the printed poly-lactic acid holder and of the Instron machine itself should also be taken into consideration. 

Finally, further characterisation can be performed towards the evaluation for safe usage such as thermal characterisation, cyclic loading, and many other in-situ methods for electronic materials. Quantifying the increased surface area in CNC–CNT films will enable comparison to conventional electrodes. Cross-sectional microscopy of the flexible capacitors will enable pre- and post-mortem interface microstructure characterisation. 

## 5. Conclusions

This work reports a novel class of biobased nanocomposites for use in electronic applications. Simple and safe composites of CNC–protein were fabricated in nominal conditions, taking advantage of CNC’s ability to self-assemble in strong, flexible, transparent and highly ordered thin films. The CNC–protein nanocomposites made covered a spectrum of dielectric permittivity from *ε_r_* = 4 to 50. Electronically conductive films were also fabricated by dispersing CNTs in CNC. The stacking of the different films fabricated enabled the fabrication of simple, cheap, and degradable capacitors. This preliminary report paves the way for the characterisation of CNC–protein composites as platform solutions for use in dielectric applications. The limits of the results presented were discussed and routes for further characterisation were suggested in order to account for the complexity of the composites, such as anisotropy, and the influence of residual water due to cellulose’s hygroscopic nature. 

This paper is the first to introduce cheap, scalable proteins as additives for the enhancement of the electronic properties of CNC-based films. The raw materials used are quasi-agnostic to location, which could also cut down transportation and geopolitical constraints on the supply chain.

## Figures and Tables

**Table 1:** Average transmittance of the various samples in the visible range (400 to 800 nm). The graphical results including the UV range are available in the supplementary information.

Sample	Average Optical Transmittance over the Visible Range (%)
CNC	84.64 ± 1.82
BSA	83.15 ± 1.14
CBS BSA	76.09 ± 2.23
ChickP G	49.26 ± 1.31
Yeast protein	16.81 ± 1.31
ChickP S	61.73 ± 1.18
Aquafaba	85.06 ± 1.34
Pea	81.77 ± 2.15
Sunflower	73.66 ± 7.46
Canola	74.67 ± 2.12
Zein	59.56 ± 3.02

**Table 2:** Discharge of devices after 5 min charging under 10 V applied voltage.

Sample	Voltage Post Charging (V)	Predicted Time to Reach 1.5 V	Predicted Time to Full Discharge (0 V)
CNC	1.86	13.5 s	3.31 h
CBS	2.21	6.24 min	6.3 days
CBS + BSA	2.32	6.3 min	14.5 days
CNC + BSA	2.30	3.36 min	3.5 days
Aquafaba	2.62	11.12 min	4.1 days
Sunflower	2.45	8.97 min	6.7 days
Pea	2.70	15.96 min	8.4 days
Canola	2.47	6.89 min	6.9 days

## Data Availability

All data presented in this work is made available in the supplementary info.

## Supplementary Materials

The following supporting information can be downloaded at: https://www.mdpi.com/article/10.3390/nano13152258/s1, Figure S1: printed parallel plate capacitor holder; Figure S2: UV transmittance spectra of CNC-based films.; Figure S3: Visible spectra of CNC-based films; Figure S4: visual comparison of CNC vs. CNC Sunflower film; Figure S5: Tensile testing graphs of CNC-based films; Figure S6: Bending of a CNC–CNT film; Figure S7: FTIR spectra of CNC-based films; Figure S8: Discharge of capacitors. Table S1: logarithmic fit of discharge curves.
