# Supplementary material for: Biobased Electronics: Tunable Dielectric and Piezoelectric Cellulose Nanocrystal—Protein Films
Source: Nanomaterials (Basel). 2023 Aug 6;13(15):2258. doi: 10.3390/nano13152258 (PMC10421335; doi:10.3390/nano13152258)
Supplement: Supplementary file 1 [file nanomaterials-13-02258-s001.zip › molecules-2531589-supplementary.pdf]

Supplementary Information

Biobased Electronics: Tunable Dielectric and Piezoelectric Cellulose Nanocrystal–Protein Films

Daniel Voignac, Shylee Belsey, Elisabeth Wermter, Yossi Paltiel and Oded Shoseyov

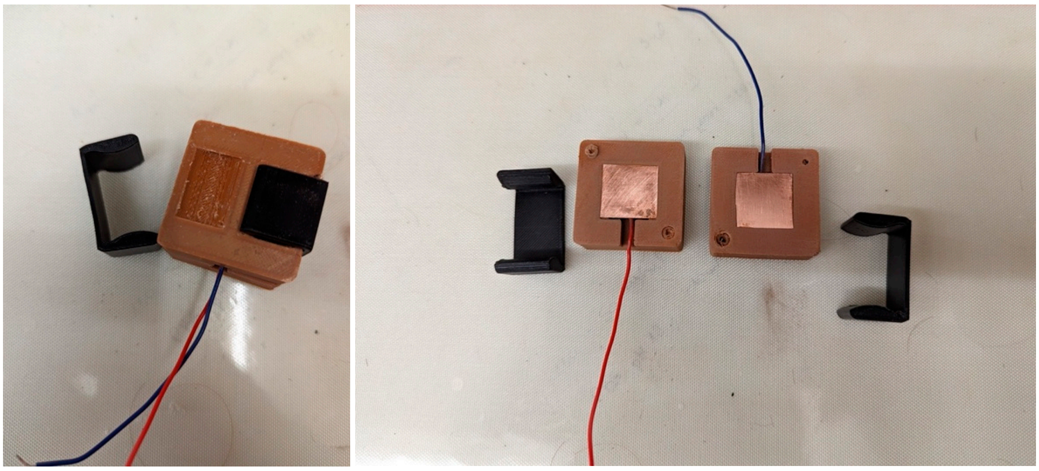

*Figure S1 Setup design to generate a parallel plate capacitor. 3D printed from PLA in which pure Cu plates of 10 mm*10 mm were embedded and soldered to a Cu cable on their backside. Printed clips allowed to apply constant force on the film. This device was used to collect data on the dielectric permittivity and piezolectric effect. Thanks are due to Omer Shoseyov for designing.*

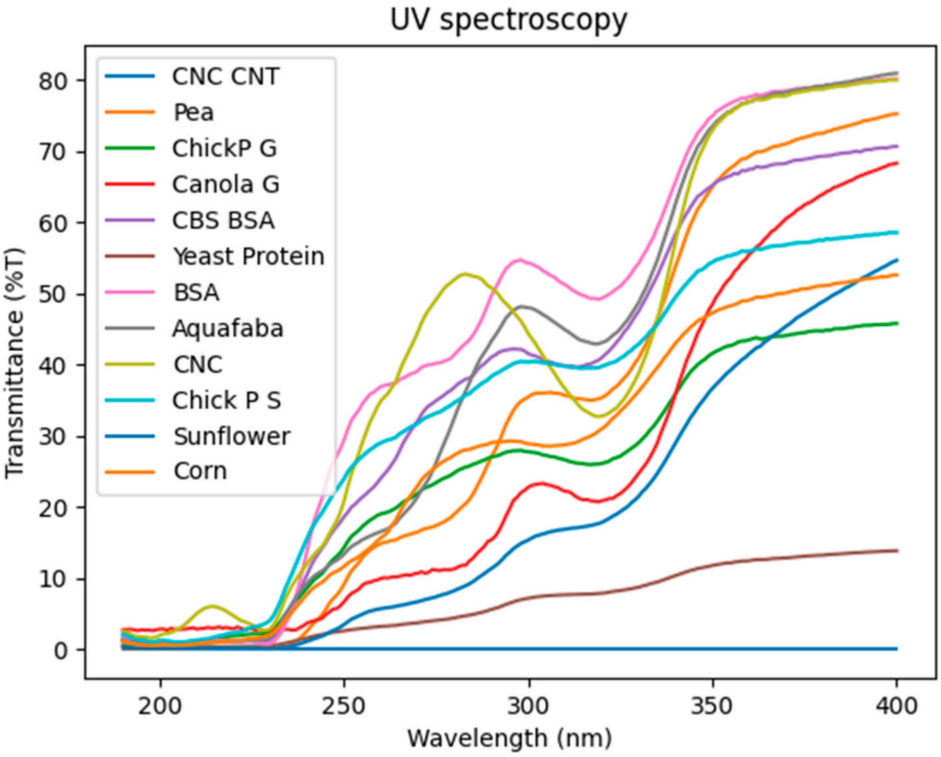

Figure S1 UV Spectroscopy of the various films prepared. CNC has a transmittance peak in the UV around 275nm which is shifted closer to 300 nm for the CNC-protein films. The CNC CNT does not transmit any light across UV.

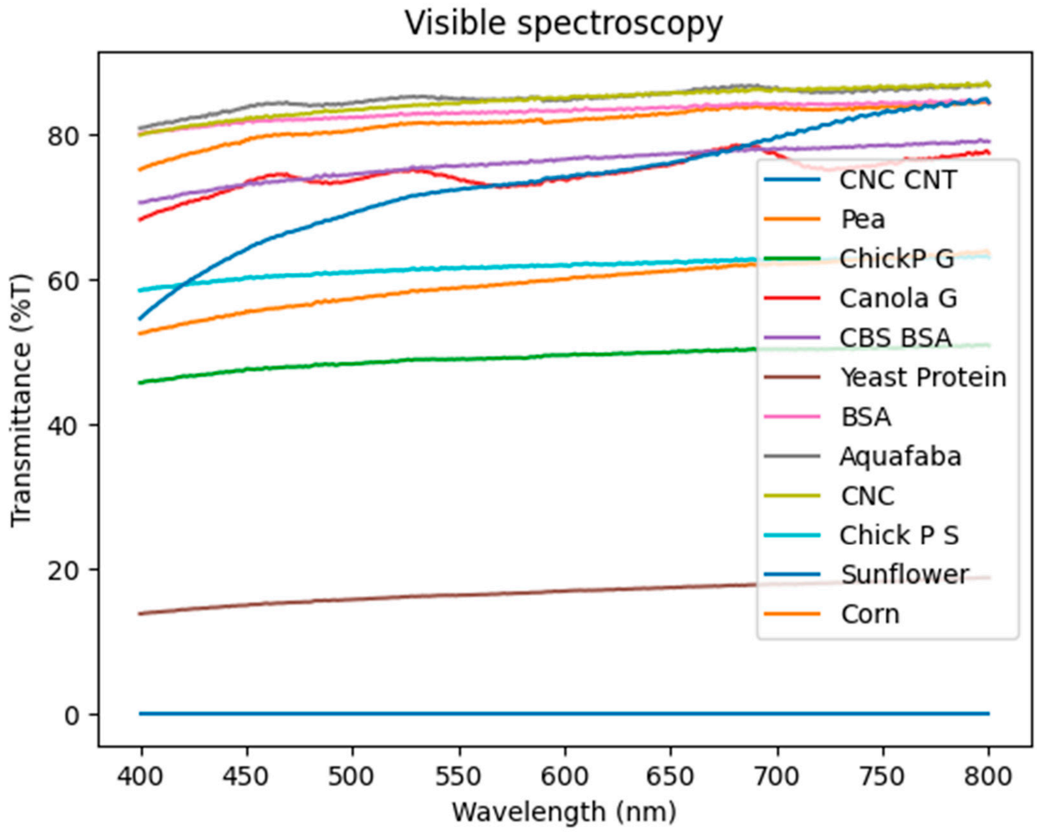

Figure S2 Visible spectroscopy of the various films prepared. Most films exhibit constant transmittance across the visible range. Sunflower is the only film which has significantly less transmittance in the colder region of the spectrum. The CNC CNT does not transmit any light across the visible range.

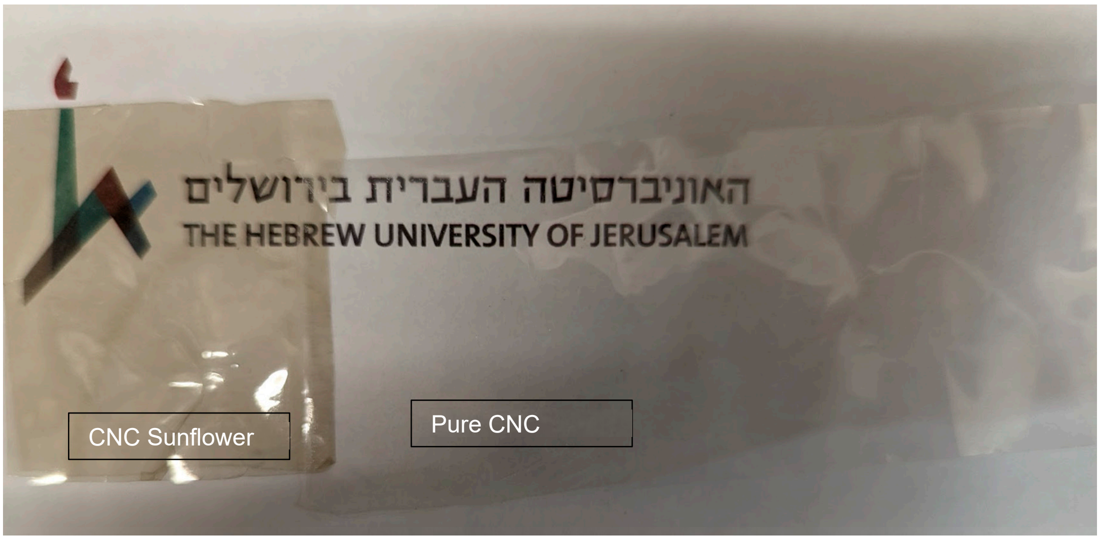

Figure S3 Side-by-side comparison of a CNC-Sunflower (left) vs. a pure CNC film (right)

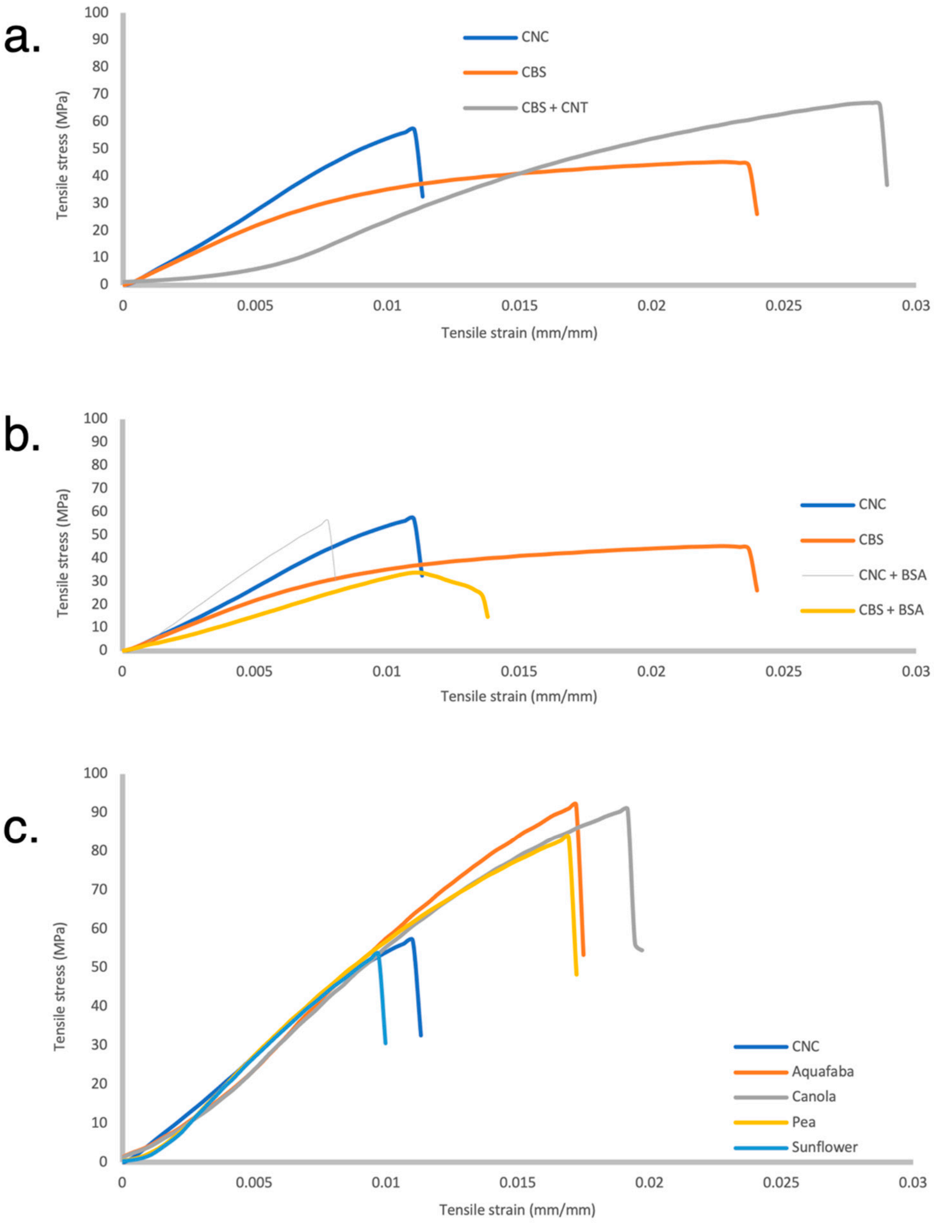

Figure S5 Strain-stress graphs for the various films prepared. (a) CNT and crosslinked reference (b) BSA containing films (c) Plant based protein films

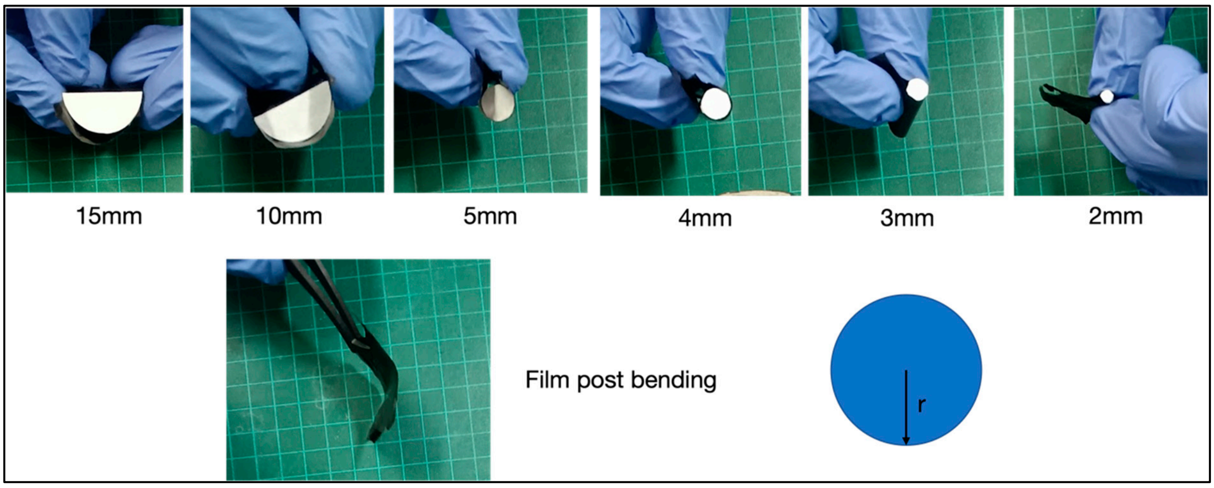

Figure S6 Illustration of the bending radius evaluation. Here a CNC-CNT film is bent along 3D printed rods of decreasing radius.

Chemical analysis

The cellulose film shows a broad peak at 3330 cm^{-1}, corresponding the O-H stretching vibration of the hydroxyl groups in the cellulose, and bonded H-molecules in the cellulose. Another peak is located at 2897 cm^{-1} and indicates the C-H stretching within the polysaccharide. Further peaks can be found in the "fingerprint region", where at 1420 cm^{-1} a ring stretching vibration and C-H in-plane bending occured, which is characteristic for cellulose. Peaks at 1314 and 1030 cm^{-1} indicate the stretching vibrations of C-O bonds, and the peak at 1110 cm^{-1} represents vibrations of the ring structure of cellulose. The $O\text{-}CH_3$ bond in cellulose can be identified in the peak at 981 cm^{-1}. The CNC crosslinked by BTCA and SHP features a peak at 1718 cm^{-1} when compared to the pure CNC. By adding animal proteins, there is hardly any change compared to the reference, only the intensity of the peaks at 981 cm^{-1} are slightly affected. Films with added plant proteins show a further peak at approximately 1654 cm^{-1}, which can be attributed to the amides of the proteins. Furthermore, an amplification of the peaks at 3330 cm^{-1} and 1030 cm^{-1} is seen due to the addition of proteins.

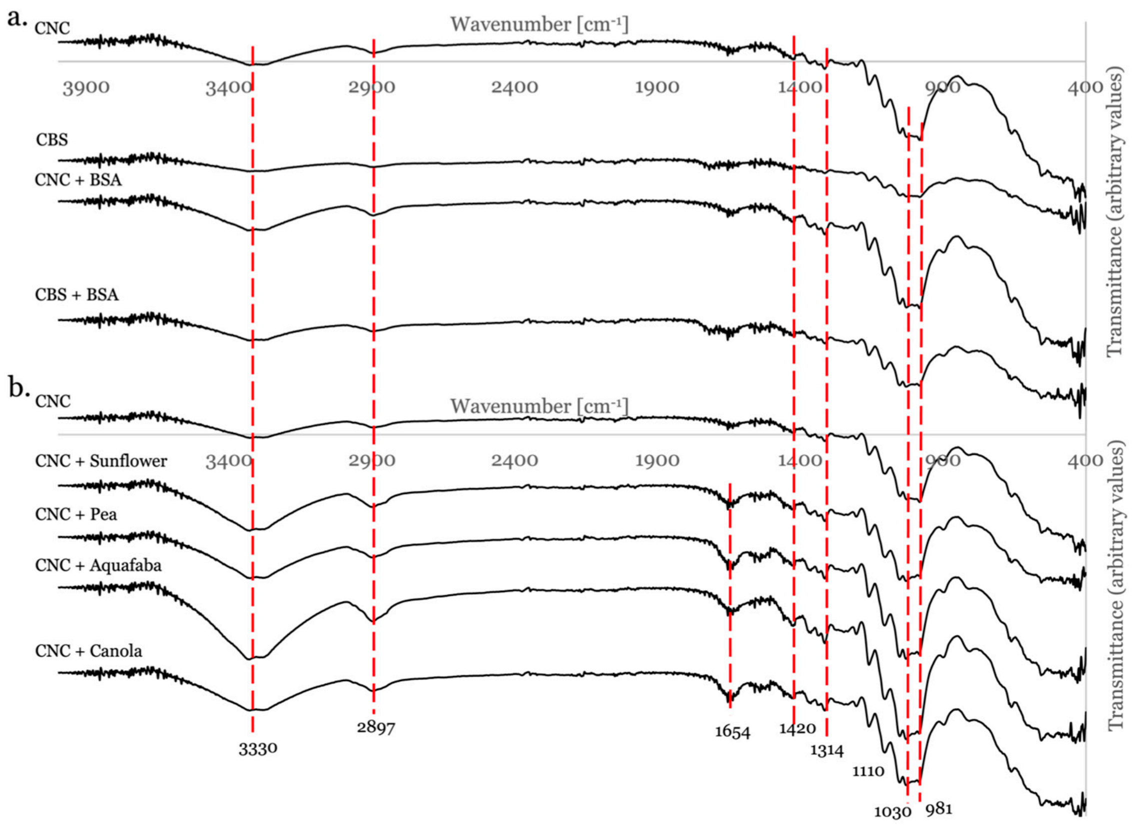

Figure S7 ATR FT-IR Spectroscopy of different films with arbitrary values for the y-axis. a. FT- IR spectrum of CNC compared to CNC-based films of animal origin. b. FT-IR spectrum of CNC compared to CNC-based films of plant origin. The dashed red lines mark common relevant peaks and the corresponding values.

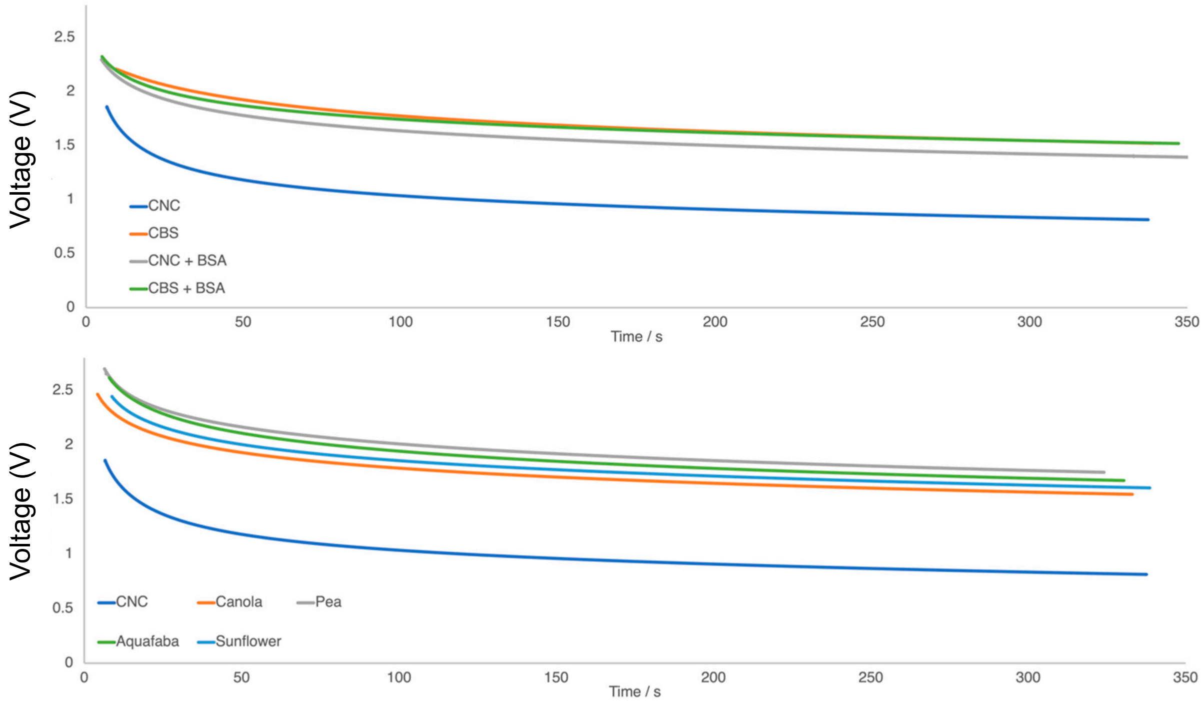

Figure S4 Discharging graphs for the capacitors built. After 5 min charging under 10 V.

Table S1 Logarithmic fits for the discharge curve after poling the capacitors resulting in the values of table 2.

	CNC	CBS	CNC + BSA	CBS + BSA
y	-0,22ln(x) + 2,0742	-0,206ln(x) + 2,7207	-0,205ln(x) + 2,588	-0,185ln(x) + 2,598
R^2	0,98	0,999	0,9988	0,9993
	CNC + Canola	CNC + Pea	CNC + Aquafaba	CNC + Sunflower
y	-0,206ln(x) + 2,7411	-0,227ln(x) + 3,0583	-0,239ln(x) + 3,0542	-0,215ln(x) + 2,8519
R^2	0,9996	0,9992	0,9982	0,9984
